# Labelling and quality of antimicrobial products used in chicken flocks in the Mekong Delta of Vietnam

**DOI:** 10.1002/vms3.189

**Published:** 2019-08-02

**Authors:** Nguyen Thi Phuong Yen, Doan Hoang Phu, Nguyen Van Cuong, Bach Tuan Kiet, Be Vo Hien, Pawin Padungtod, Dinh Bao Truong, Guy E. Thwaites, Juan J. Carrique‐Mas

**Affiliations:** ^1^ Oxford University Clinical Research Unit Hospital for Tropical Diseases Ho Chi Minh City Vietnam; ^2^ Sub‐Department of Animal Health and Production Cao Lanh Dong Thap Province Vietnam; ^3^ Emergency Center for Transboundary Animal Diseases Food and Agriculture Organization of the United Nations Hanoi Vietnam; ^4^ Faculty of Animal Science and Veterinary Medicine Nong Lam University Ho Chi Minh Vietnam; ^5^ Nuffield Department of Medicine Oxford University Headington Oxford United Kingdom

**Keywords:** animal production, antimicrobials, poultry, veterinary, Vietnam

## Abstract

**Background:**

The Mekong Delta of Vietnam is a hotspot of antimicrobial use (AMU), but there is no information on the quality of the labelling and strength of antimicrobial products used in poultry production.

**Methods:**

Based on a large random sample of farms, we identified the 20 most used antimicrobial products in the area, and investigated their antimicrobial active ingredient (AAI) content by UPLC‐MS/MS (91 analytical tests).

**Results:**

Only 17/59 (28.8%) batches contained all AAIs within 10% of the declared strength. Worryingly, 65.0% products provided in their label preparation guidelines for both therapeutic and prophylactic use. Withdrawal times for both meat and eggs were stated in 8/20 (40%) products.

**Conclusion:**

Results highlight deficiencies in quality and labelling contents that undermine authorities’ efforts to discourage inappropriate use of antimicrobials.

## Abstract 2

We analysed the quality (strength) and labelling of 20 most commonly used antimicrobial products from a large representative survey of chicken farms in the Mekong Delta of Vietnam. The study identified a large proportion of products (71.9%) containing quantities of active ingredient outside the permitted range, as well while 65% of these products explicitly advised on prophylactic use (i.e. to prevent disease).

## Graphical abstract

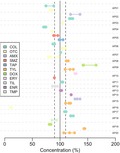

## 1 INTRODUCTION

Antimicrobials are widely used in animal production, both to prevent and to treat diseases. In some countries, antimicrobials are also added to commercial feed formulations to promote rapid growth (Page & Gautier, 2012). It has been estimated that in African countries about 50% of antimicrobials available in the market correspond to non‐standard and non‐registered veterinary medicines (Clifford et al., 2018). There is a concern that inadequate formulation of these products may lead to exposure to sub‐therapeutic levels of antimicrobials, therefore promoting resistance among bacterial populations (Nwokike, Clark, & Nguyen, 2018). Recent studies on the quality of antimicrobial products used in shrimp and catfish farming in Vietnam indicated that only ~8% and ~29% products contained an AAI within ±10% (accepted level of variation) (Phu, Phuong, Scippo, & Dalsgaard, 2015; Tran, Tran, Phan, & Dalsgaard, 2018). Globally, the quantity of antimicrobials used in chicken production is estimated at 138.0 doses/1,000 animal‐days [inter quartile range (IQR) 91.1–438.3], a higher amount than AMU in the two other major terrestrial food animal species (pig and cattle) (Cuong, Padungtod, Thwaites, & Carrique‐Mas, 2019). Previous studies have reported exceptionally high levels of antimicrobial use (AMU) in chicken farms in the Mekong Delta region of Vietnam (Carrique‐Mas et al., 2015; Carrique‐Mas et al., 2019; Cuong et al., 2019; Nguyen et al., 2016). However, there are currently no published data on the quality of antimicrobial products used in these farming systems. We investigated the labelling and strength of AAIs of the most commonly used products in representative chicken farms in the Mekong Delta of Vietnam.

Antimicrobial products were identified from a survey of 102 randomly selected farms raising meat chickens in Dong Thap province from November 2016 to March 2018. A total of 203 flocks raised in those farms with a completed full cycle of production were included in the study (Carrique‐Mas & Rushton, 2017; Cuong et al., 2019). All flocks consisted of native breed chickens raised over a median period of 18 [Interquartile Range 16‐20] weeks, with birds typically raised using all‐in‐all‐out system. At the beginning of the project, farmers were given purposefully designed diaries to record their AMU, as well as containers where farmers were asked to store all packages of antimicrobials. A team of trained animal health workers visited each farm four times during each production cycle to review the collected data. The 20 most frequently used antimicrobial products were identified. Three different batches of each product were purchased from veterinary drug shops within the province of Dong Thap. The 20 most commonly used antimicrobial‐containing products (defined as the proportion of flocks using) were identified, and information on strength on AAIs, species target, prophylactic/therapeutic indication, and withdrawal times for meat and egg productions was compiled. The products’ contents were tested (single blinded) for the presence and strength of the AAIs declared in the label at an accredited laboratory (Center for Analysis Service of Experiment, Ho Chi Minh City, ISO 9001:2008 accredited) using Ultra High Performance Liquid Chromatography coupled to tandem Mass Spectrometry (UPLC‐MS/MS). Three aminoglycoside antimicrobials (gentamicin, neomycin and streptomycin) were not investigated. For colistin, the number of International Units (IU) indicated in the label was converted to miligrams. Results were expressed as a percent of the declared strength indicated in the label (percent content). The inter‐batch variability (in relation to the overall variability) was investigated by fitting a null random effects model with product fitted as a random effect and percent content as the outcome using lme4 package and R software.

The 20 products identified were marketed by nine different companies, and all except one (a French company selling product AB008) were Vietnamese (Table 1). All products were formulated for oral administration: Nineteen (95%) were powder‐based formulations and one (5%) was a liquid solution. Five (25%) products contained a single antimcrobial and 16 (75%) a mixture of two antimicrobials. In order to investigate the inter‐batch variability, three batches of 19 products and two batches of one product (AB051) were investigated, making a total of 91 analytical tests (Table 1).

**Table 1:** Characteristics of 20 antimicrobial products investigated, sorted by the number of flocks where they were used

Product code	No. flocks used (%)	Company	Package content	Target species	Declared strength (%=g/100 g product)	Indication	Product description	Withdrawal time indicated (meat, eggs)
AP01	34.5	A	100 g	Chickens, ducks	OTC 1.5%; COL 0.07%	Prophylactic, therapeutic	Antimicrobial mixed with vitamins	Meat
AP02	14.8	F	20 g	Poultry, ruminants, pigs, horses	AMX 10%; COL 2%	Therapeutic	Antimicrobial only	Meat, eggs
AP03	14.8	A	100 g	Poultry	OTC 10%; STR 5%	Therapeutic	Antimicrobial only	Meat
AP04	11.8	G	50 g	Poultry	OTC 4%; COL 2%	Prophylactic, therapeutic	Antimicrobial mixed with vitamins	No
AP05	11.8	G	100 g	Poultry, ruminants, pigs	TAP 6%; SMZ 5%	Prophylactic, therapeutic	Antimicrobial only	Meat
AP06	10.8	D	100 g	Poultry, ruminants, pigs	OTC 5%; COL 0.017%	Prophylactic, therapeutic	Antimicrobial mixed with vitamins	Meat, eggs
AP07	8.4	B	100 g	Poultry, pigs	OTC 7%; COL 0.98%	Prophylactic, therapeutic	Antimicrobial only	Meat, eggs
AP08	8.4	G	100 g	Poultry, ruminants, pigs	TYL 7%; GEN 3.5%	Prophylactic, therapeutic	Antimicrobial mixed with minerals	Meat, eggs
AP09	7.4	H	100 g	Poultry, ruminants, pigs	DOX 20%; TYL 10%	Prophylactic, therapeutic	Antimicrobial only	Meat, eggs
AP10	5.9	G	100 g	Poultry	ERY 6%; SMZ 10%	Prophylactic, therapeutic	Antimicrobial only	Meat
AP11	5.4	C	50 g	All animal species	GEN 6%; COL 2.44%	Therapeutic	Antimicrobial mixed with vitamins, anti‐inflammatory	Meat, eggs
AP12	5.4	G	100 mL	Poultry, ruminants, pigs	TIL 25%	Prophylactic, therapeutic	Antimicrobial only	Meat
AP13	5.4	H	100 g	Poultry, ruminants, pigs	ENR 5%	Prophylactic, therapeutic	Antimicrobial mixed with expectorant, analgesic	Meat
AP14	4.9	I	100 g	Ducks, Muscovy ducks	ENR 5%	Prophylactic, therapeutic	Antimicrobial only	Meat
AP15	4.4	I	100 g	Poultry	AMX 10%; TYL 10%	Prophylactic, therapeutic	Antimicrobial mixed with expectorant, analgesic	Meat
AP16	3.9	A	100 g	Poultry, ruminants, pigs	OTC 1%	Not explicit	Antimicrobial mixed with vitamin, analgesic, antipyretic	Meat, eggs
AP17	3.9	I	100 g	Poultry, ruminants, pigs	NEO 6%; COL 1.46%	Therapeutic	Antimicrobial only	Meat
AP18	3.4	I	100 g	Poultry, ruminants, pigs	TMP 3%; COL 2%	Therapeutic	Antimicrobial only	Meat, eggs
AP19	3.0	E	100 g	Poultry	DOX 2.5%; TYL 2.5%	Prophylactic, therapeutic	Antimicrobial mixed with vitamin	Meat
AP20	1.0	C	50 g	Poultry, ruminants, pigs	GEN 3%; TYL 5%	Therapeutic	Antimicrobial only	Meat

Abbreviations: AMX, amoxicillin; COL, colistin; DOX, doxycycline; ENR, enrofloxacin; ERY, erythromycin; GEN, gentamicin; NEO, neomycin; OTC, oxytetracycline; SMZ, sulphametoxazole; STR, streptomycin; TAP, thiamphenicol; TIL, tilmicosin; TYL, tylosin.

Twelve different AAIs were identified in the 20 products, the most common being: colistin (8 products), oxytetracycline (6), gentamicin (2), tylosin (2), doxycycline (2), amoxicillin (2) and enrofloxacin (2). Other AAIs (trimethoprim, streptomycin, tilmicosin, erythromycin and neomycin) were contained in one product each. Six of those AAIs (colistin, gentamicin, tylosin, erythromycin, tilmicosin and neomycin) are considered to be critically important antimicrobials according to the World Health Organization (Anon 2017).

**Figure 1:**
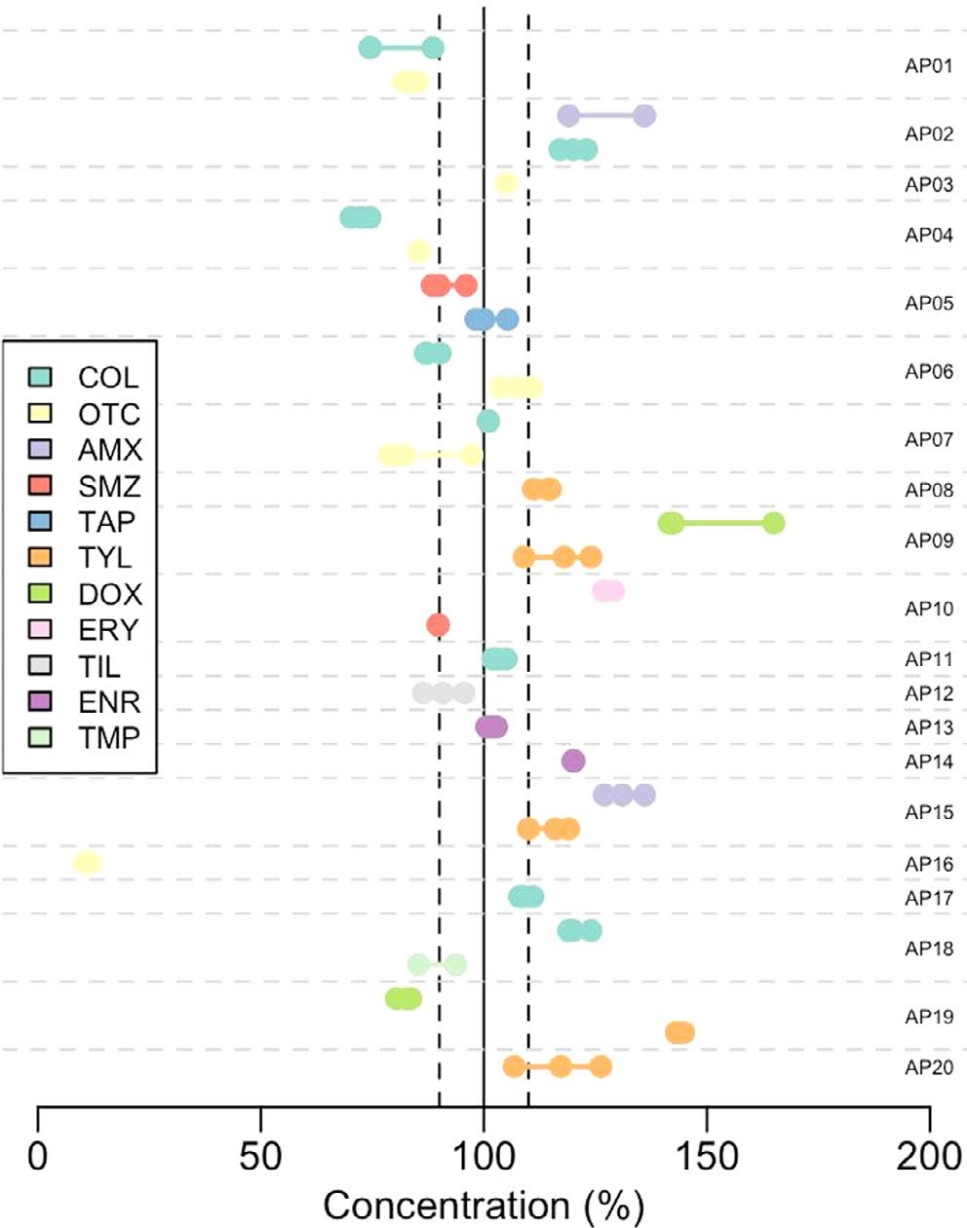
Results of the analyses of strength of antimicrobial AAIs in the 20 most commonly used products in poultry farms in the Mekong Delta of Vietnam. Products are sorted by decreasing prevalence of use by flock. Each dot across horizontal line corresponds to the results of the concentration of one AAI analysed

In six (30.0%) products the label provided an explicit indication for therapeutic administration only, 13 (65.0%) products provided an indication for both therapeutic and prophylactic use, and one (5.0%) did not include any indication. Withdrawal times for both egg and meat production were provided in the labels of eight (40.0%) products; in 11 (55.0%) products withdrawal times were indicated only for meat (but not for eggs); one product contained no indications with respect to withdrawal time. A total of 11 (55.0%) products contained only one AAI, and the remaining had other substances (including vitamins, mineral supplements and expectorants and analgesic substances). Twenty‐eight (30.8%) samples tested were within 10% of the strength declared in the label. Thirty‐four (37.4%) contained AAIs above the declared upper limit, and 27 (29.7%) below the declared lower limit. Two extreme values were observed for two AAIs: one (Product AP16) contained oxytetracycline with strength ranging from 10.3% to 11.9% and another (AB09) product had doxycycline strength ranging from 141.5% to 165.0% of the stated value (Figure 1).

In 27/91 (29.7%) of the tests conducted the AAIs had a strength below the acceptable lower limit (−10%). Unexpectedly, 34/91 (37.4%) had AAIs with strength higher than that indicated in the label. Of the 59 individual product batches investigated, only 17 (28.8%) had all their AAIs within the ±10% acceptable range. Only 3 of the 20 (15.0%) products had all batches and all their AAIs within the ±10% range. A total of 24.5% of the variance was attributed to between‐batch variation, the remainder being due to between‐product variation.

Since our study is based on a random sample of farms, we are confident that these results are representative of antimicrobial products most commonly used by poultry farmers in the Mekong Delta of Vietnam. Currently there are >10,000 licensed veterinary products in the country, of which about ~50% consist of antibacterial antimicrobial formulations (Anon 2016). This makes quality control monitoring extremely challenging, particularly in a limited‐resource setting such as Vietnam.

Quality testing of AAIs is very costly, and there is a lack of unbiased information about this issue in animal production in most countries. It has been previously estimated that one in 10 medicinal products in low‐ and middle‐income countries is substandard or falsified (Nwokike et al., 2018). Given that the identity of antimicrobials declared in the label was confirmed in all cases, we do not believe that outright falsification is a major issue here. Furthermore, ‘legal’ antimicrobials are currently very affordable in Vietnam, and two‐thirds of the products investigated had an indication for ‘prophylactic use’ in the label (normally followed by a list of bacterial diseases). This labelling openly conflicts with the animal health authorities’ efforts to discourage routine use of antimicrobials for preventing disease (Aidara‐Kane et al., 2018; Anon 2013) and sends a ‘wrong’ message to farmers (the end users), who will not be able unable to discern in the few instances that medication may be required in the absence of disease. This is particularly relevant in the context of  small‐scale farmers in many low‐ and middle‐income countries. Farmers in these settings  often do not have access to veterinary services capable of providing them with unbiased advice on AMU.

Under dosing is expected to result because of either sub‐optimal quality of the manufactured product, or inadequate preparation at the point of administration by the farmer. For most products, the guidelines for product preparation (mixing with water) for prophylaxis were about half the strength required for therapeutic purposes. There is a risk that this may increase the probability of selection of AMR in bacterial populations (Ungemach, Mueller‐Bahrdt, & Abraham, 2006). Withdrawal times for egg production were not specified in 60% of the antimicrobial products investigated. This is a concern, since these products are likely to be used both in meat and layer flocks. The observed inter‐batch variation in product quality suggests deficiencies in the mixing/packaging process, since in Vietnam most AAIs sold in Vietnam are bulk‐imported and then mixed, packaged and distributed within the country.

Based on a representative field survey, we identified the most common antimicrobial products used in poultry farming in the Mekong Delta. Results indicate variable quality results, with only 17 (28.8%) product batches containing AAIs within the acceptable ±10% range. In addition to improving quality control of veterinary medicine products, we strongly advocate for enhancing regulation and inspection of antimicrobial product labelling, crucially removing the indication for prophylactic use. In all cases, products should indicate withdrawal times for meat, eggs and milk (for products aimed at ruminants). It would be desirable to limit the access to antimicrobials of critical importance for human health for veterinary use, and therefore development of policies aiming at this should be a priority.

## CONFLICT OF INTEREST

The authors declare no conflict of interest.

## ETHICAL STATEMENT

The authors confirm that the ethical policies of the journal, as noted on the journal's author guidelines page, have been adhered to. No ethical approval was required, as this is a retail study, with no direct implications or impact on any particular subject.

## References

[vms3189-bib-0001] Aidara‐Kane, A. , Angulo, F. J. , Conly, J. M. , Minato, Y. , Silbergeld, E. K. , McEwen, S. A. , & Collignon, P. J. (2018). World Health Organization (WHO) guidelines on use of medically important antimicrobials in food‐producing animals. Antimicrobial Resistance & Infection Control, 7(1), 7. 29375825 10.1186/s13756-017-0294-9 PMC5772708

[vms3189-bib-0002] Anon . (2013). RUMA seeks to clarify the position on preventive use of antibiotics. Veterinary Record, 172, 379. 23585098 10.1136/vr.f2240

[vms3189-bib-0003] Anon . (2016). List of authorized imported antimicrobial products for animal use [in Vietnamese] Department of Animal Health, Vietnam. Available at: https://bientap.vbpl.vn//FileData/TW/Lists/vbpq/Attachments/114545/VanBanGoc_Phu%20luc%201B.%20Danh%20muc%20thuoc%20thuoc%20nhap%20khau%202016.pdf(Accessed 15 Jan 2019).

[vms3189-bib-0004] Anon . (2017). WHO critically important antimicrobials for human medicine 5^th^ revision World Health Organization, Geneva. Available at: https://www.who.int/foodsafety/areas_work/antimicrobial-resistance/cia/en(Accessed 4 Jan 2019)

[vms3189-bib-0005] Carrique‐Mas, J. , & Rushton, J. (2017). Integrated Interventions to Tackle Antimicrobial usage in animal production systems: The ViParc Project in Vietnam. Frontiers in Microbiology, 8, 1062. 28659887 10.3389/fmicb.2017.01062 PMC5468401

[vms3189-bib-0006] Carrique‐Mas, J. J. , Trung, N. V. , Hoa, N. T. , Mai, H. H. , Thanh, T. H. , Campbell, J. I. , … Schultsz, C. (2015). Antimicrobial usage in chicken production in the Mekong Delta of Vietnam. Zoonoses and Public Health, 62, 70–78. 25430661 10.1111/zph.12165

[vms3189-bib-0007] Carrique‐Mas, J. , Van, N. T. B. , Van Cuong, N. , Truong, B. D. , Kiet, B. T. , Thanh, P. T. H. , … Choisy, M. (2019). Mortality, disease and associated antimicrobial use in commercial small‐scale chicken flocks in the Mekong Delta of Vietnam. Preventive Veterinary Medicine, 165, 15–22. 30851923 10.1016/j.prevetmed.2019.02.005 PMC6418316

[vms3189-bib-0008] Clifford, K. , Desai, D. , da Costa, C. P. , Meyer, H. , Klohe, K. , Winkler, A. S. , … Zaman, M. H. (2018). Antimicrobial resistance in livestock and poor quality veterinary medicines. Bulletin of the World Health Organization, 96(9), 662. 30262949 10.2471/BLT.18.209585 PMC6154060

[vms3189-bib-0009] Cuong, N. , Padungtod, P. , Thwaites, G. , & Carrique‐Mas, J. (2019). Antimicrobial usage in animal production: a review of the literature with a focus on low‐and middle‐income countries. Antibiotics, 7(3), 75. 10.3390/antibiotics7030075 PMC6164101 30111750

[vms3189-bib-0010] Cuong, C. , Phu, D. H. , Van, N. T. B. , Truong, B. D. , Kiet, B. T. , Vo, H. B. , … Carrique‐Mas, J. J. (2019). High resolution monitoring of antimicrobial consumption in Vietnamese small‐scale chicken farms highlights discrepancies between study metrics. Frontiers in Veterinary Science, 6, 174. 31294033 10.3389/fvets.2019.00174 PMC6598194

[vms3189-bib-0011] Nguyen, N. T. , Nguyen, H. M. , Nguyen, C. V. , Nguyen, T. V. , Nguyen, M. T. , & Thai, H. Q. (2016). Use of colistin and other critical antimicrobials on pig and chicken farms in southern Vietnam and its association with resistance in commensal *Escherichia coli* bacteria. Applied and Environmental Microbiology, 82, 3727–3735. 27084016 10.1128/AEM.00337-16 PMC4907207

[vms3189-bib-0012] Nwokike, J. , Clark, A. , & Nguyen, P. P. (2018). Medicines quality assurance to fight antimicrobial resistance. Bulletin of the World Health Organization, 96, 135–137. 29403117 10.2471/BLT.17.199562 PMC5791778

[vms3189-bib-0013] Page, S. W. , & Gautier, P. (2012). Use of antimicrobial agents in livestock. Revue Scientifique et Technique (International Office of Epizootics), 31, 145–188. 22849274 10.20506/rst.31.1.2106

[vms3189-bib-0014] Phu, T. M. , Phuong, N. T. , Scippo, M. L. , & Dalsgaard, A. (2015). Quality of antimicrobial products used in striped catfish (*Pangasianodon hypophthalmus*) Aquaculture in Vietnam. PLoS ONE, 10, e0124267. 25897517 10.1371/journal.pone.0124267 PMC4405571

[vms3189-bib-0015] Tran, K. C. , Tran, M. P. , Phan, T. V. , & Dalsgaard, A. (2018). Quality of antimicrobial products used in white leg shrimp (*Litopenaeus vannamei*) aquaculture in Northern Vietnam. Aquaculture, 482, 167–175.

[vms3189-bib-0016] Ungemach, F. R. , Mueller‐Bahrdt, D. , & Abraham, G. (2006). Guidelines for prudent use of antimicrobials and their implications on antibiotic usage in veterinary medicine. International Journal of Medical Microbiology, 296, 33–38. 16520092 10.1016/j.ijmm.2006.01.059

